# Information needs of patients with chronic diseases and their relatives for web-based advance care planning: a qualitative interview study

**DOI:** 10.1186/s12904-021-00770-x

**Published:** 2021-05-30

**Authors:** Doris van der Smissen, Judith A. C. Rietjens, Lisette van Gemert-Pijnen, Sandra van Dulmen, Agnes van der Heide, Ida J. Korfage

**Affiliations:** 1 Department of Public Health, Erasmus MC, University Medical Center Rotterdam, P.O. Box 2040, 3000 CA Rotterdam, the Netherlands; 2 Department of Psychology, Health and Technology, University of Twente, Enschede, the Netherlands; 3 Department of Primary and Community Care, Radboud university medical center, Nijmegen, the Netherlands; 4 Nivel (Netherlands institute for health services research), Utrecht, the Netherlands

**Keywords:** Advance care planning, Chronic disease, Web-based information, Health communication, Interviews, Information needs

## Abstract

**Background:**

Advance care planning (ACP) enables persons to identify preferences for future treatment and care, and to discuss, record and review these preferences. However, the uptake of ACP among patients with chronic diseases is relatively low. Web-based ACP programs can support patients and their relatives in ACP. However, information needs of patients and their relatives for ACP are unknown. The aim of this study is to explore information needs of patients with chronic disease and their relatives for web-based ACP.

**Methods:**

We conducted semi-structured interviews with patients with chronic diseases and relatives at their home or at the study center. In three cases, the patient and relative were paired since they preferred to be interviewed together. We asked about information they would search for when to start with ACP, where they would search for information, what search terms they would use on the Internet, and what content and information they would consider important on an ACP website. The interviewer asked participants to clarify their responses during the interview. We used thematic analysis to analyze the interviewees’ responses.

**Results:**

We interviewed nine patients with different chronic diseases including amyotrophic lateral sclerosis (ALS), multiple sclerosis (MS), chronic obstructive pulmonary disease (COPD) and kidney diseases, and seven relatives, namely partners or (adult) children. The interviewees were aged 24 to 80 years, nine were female and seven were male. Both patients with a chronic disease and relatives mentioned comparable information needs. Many interviewees indicated they would use the Internet to search for information about ACP. Mentioned search terms were “advance care planning”, “treatment plan”, “disease trajectory” and names of patient associations. Information needs concerned their disease trajectory and quality of life, medical treatment decisions, practical support in arranging care, the concept of ACP and guidance in ACP, communication of treatment and care preferences, peer support of others with chronic diseases, and information for relatives. Many appreciated encouragement of their healthcare providers to take a pro-active role in ACP.

**Conclusions:**

We conclude that information needs for ACP included guidance in ACP, support in making decisions about medical treatment, and practical support in arranging care. We recommend adapting web-based ACP information to the information needs of patients and their relatives to increase its findability, uptake and usefulness.

**Supplementary Information:**

The online version contains supplementary material available at 10.1186/s12904-021-00770-x.

## Background

Advance care planning (ACP) enables persons to define goals and preferences for future medical treatments and care, to discuss these with healthcare professionals and relatives, and to record and review these if appropriate [1]. Although ACP can be important at any stage of life, it can be more relevant and targeted for persons with chronic conditions or elderly people, since their health condition is more likely to deteriorate [1].

Studies showed that patients and relatives considered timely talking about treatment preferences at the end of life important [2–4], and while policies increasingly encourage patient engagement in ACP in countries such as the Netherlands, the United States and the United Kingdom, its uptake is relatively low [1, 5, 6]. A study among the Dutch general population in 2013 showed that 70% (1372/1960) had thought about issues related to medical decision making at the end of their life [7]. However, only 13% (255/1960) had discussed their preferences with healthcare professionals: 0.3% discussed this often, 3% sometimes, and 9% seldomly [7]. Overall, 21% (412/1960) preferred information on end-of-life decision making, of which 54% would search for information on the Internet and 69% would ask their general practitioner [7]. A study in Canada among nursing home residents and relatives showed that they perceived ACP terminology as unfamiliar and difficult to understand [8].

Providing information about ACP may support patients’ awareness of its importance and of options for its application [9]. Public campaigns have been conducted to reach those aims and to encourage persons to think about preferences for future treatment and care [10]. Ideally, ACP information should focus on topics that patients consider important. Web-based ACP programs have been shown to be promising to support patients in ACP, by providing information about ACP and support in communication about treatment and care preferences with doctors and relatives [11]. An example of a web-based program for ACP is ‘PREPARE’, which has been shown to support patients in ACP [12, 13]. Web-based ACP programs may also be relevant during the COVID-19 pandemic. For instance, in Colorado the number of users of an online ACP portal tool went from 418 in one month pre-COVID to 1037 in the first month and 815 in the second month of the COVID pandemic [14].

However, little is known about information needs of patients and relatives for ACP in general nor for web-based ACP information specifically. Therefore, the aim of this study is to explore patients’ and relatives’ information needs for web-based ACP. Insight into these information needs will be used in the development of a web-based ACP program in the Netherlands, aimed at supporting patients to think about their treatment and care preferences, to discuss these with relatives or healthcare professionals, and to record preferences in an advance directive.

## Methods

We explored information needs for ACP through semi-structured interviews with patients with chronic disease and their relatives. We aimed to recruit participants with a chronic disease, such as chronic obstructive pulmonary disease (COPD), multiple sclerosis (MS) and cancer. Furthermore, we aimed to recruit relatives with different types of relationships to patients. We used purposive sampling by selecting a comparable number of men and women, with diverse educational backgrounds, living in different areas of the Netherlands. We approached various patient organizations to invite patients with a variety of chronic diseases.

Participants were approached via phone or email by one of the authors (DS); they received information about the study and they were asked for consent to participate. Semi-structured interviews were conducted by DS at a location of the participants’ preference, namely participants’ homes (10 interviews) or at the study center (one interview), and in a care facility for COPD patients (two interviews). In three cases, a patient and a relative preferred to be interviewed together as a couple. After the interviewer’s (DS) explanation of the goal of the study, the participants gave written informed consent. The interviewer provided the respondents with the following definition of ACP: “ACP is a process which enables persons to think about their preferences for future medical treatment and care, and to discuss, record and review these if appropriate” [1]. Subsequently, the interviewer asked the interview questions, see Table 1. The questions were pilot tested with two women, without chronic disease, aged 28 and 30 years. The interviewer summarized answers of participants during the interview to ensure the answers were correctly understood. The interviewer asked participants to clarify their responses during the interview. This study was approved by the Medical Research Ethics Committee of the Erasmus University Medical Center on 11 August 2017 [MEC-2017-456]. The interviewer (DS) has an MSc degree and was educated to conduct quantitative and qualitative research. During the interviews the interviewer aimed to not express personal views. Participants had no previous knowledge about the study or the interviewer.

**Table 1:** Interview questions

(1) What information would you search for when you (or: your relative) would start with ACP? (2) Where would you search for ACP information? (3) Which search terms would you use when you would search for information about ACP on the Internet? (4) What content and information would you consider important on a website for ACP?	

### Analysis

The interviews were audio recorded and transcribed verbatim without any identifiers. We performed an inductive thematic analysis, using open, axial and selective coding [15]. DS read the transcripts from the interviews line by line and relevant segments were coded (open coding). Next, using the constant comparative method [15], these open codes were organized into initial themes, and based on these initial themes an initial coding tree was developed (selective coding). Ultimately, we organized the coded fragments into seven key information needs for ACP. In an iterative process, all steps and findings were discussed by DS, IK, JR and AH.

The goal of the study was to explore ACP information needs. Although we had a small sample size, code saturation was reached on a conceptual level (no new themes emerged from the interviews) [15]. Therefore, we decided to stop recruitment. We did participant checking with a selection of participants (1 patient and 2 relatives). These participants provided feedback during a presentation of the findings and agreed with the themes that emerged from the interviews. We followed the COmprehensive consolidated criteria for REporting Qualitative research (COREQ) [16].

## Results

### Sample characteristics

We interviewed nine patients with different chronic diseases, namely with amyotrophic lateral sclerosis (ALS) (one patient), multiple sclerosis (MS) (one patient), chronic obstructive pulmonary disease (COPD) (2 patients), kidney diseases (3 patients), muscular dystrophy (one patient) and stroke (one patient). Furthermore, we interviewed seven relatives of patients with chronic diseases, namely 6 partners in total of patients with kidney disease, cancer, dementia, muscular dystrophy, stroke, and acquired brain injury. In addition, we interviewed one person with a parent with cancer. The interviewees were nine women and seven men from different parts of the Netherlands, with different ages ranging from 24 to 80 years. The interviews lasted approximately 60 min.

### Thematic analysis

Both patients with chronic disease and relatives addressed comparable information needs, and they considered ACP and information provision about it important. Most patients and relatives would mainly search for ACP information on the Internet, for example through Google. They would consult websites of patient organizations, their healthcare professionals, their hospital or governments.

Seven main themes were identified when respondents were asked about their information needs. Representative quotations were chosen to illustrate these themes and subthemes. The coding tree with the main themes/information needs and sub themes/information needs is presented in Appendix A.

#### Disease trajectory and quality of life

The interviewees valued information about the disease process, and how to cope with it. Furthermore, the interviewees would look for support in accepting the disease. Topics considered as relevant were thinking about what is important in life, quality of life, and the impact of their chronic disease on their life. Search terms included: “What is [the disease]”; “Progression of [the disease]”; “How to live with [the disease]”; and “Consequences of [the disease] on my life”.

*Relative 1 (aged 61), when asked about what content and information he/she would consider important on a website for ACP. Subtheme “Thinking about what is important in life and about quality of life”: “The questions that people want answers to, I think that these are questions that are very close to them. And those very big life questions about whether or not to resuscitate, or suppose that I am in a coma or you name it... what happens to me then... I think it is much more about the quality of life. Quality of life has much more to do with what I can do on a daily basis, what does my day look like, what I can do, what I cannot do.”*

#### Support in medical treatment decisions

The interviewees preferred information on the impact of treatments on their life, and they wanted support in making treatment decisions. The interviewees mentioned that information about end-of-life care, cardiopulmonary resuscitation and non-treatment should be provided. Search terms included: “Hospital”; “Treatments”; “Treatment plan”; “Impact of the treatment” and “Information about euthanasia”.

*Relative 3 (62 years), subtheme “Support in making treatment decisions”: “Well, I think it is good to ask people whether they want to be resuscitated when they would be in a certain disease stage, or at an older age. It would be good to provide information about what this entails. (…) Often people don’t know the risks of resuscitation. When people receive this information, they can make a conscious decision about this. It is also important to provide information on mechanical ventilation and about what this is, how this is applied and what the effects are. This information should be described in easy language.”*

#### Practical support in arranging care

The interviewees preferred practical information on how to arrange their future care and housing. They preferred information about where and how to get support and reliable information about their disease and care. Some interviewees would look for financial information, such as the possible costs of healthcare or financial support options. Search terms included: “Home care”; “Care plan”; “Care tools”; and “Practical information”.

*Patient 2 (age 61), subtheme “Practical information on arranging care and housing”: “I think addressing people’s values on the website ​​is already very important. But I think a topic like “living at home” is also important, for example, how can you adapt your home? Can you remove thresholds in your house, and are you prepared to adapt your house if your health condition worsens? I think that is often a larger problem to people.”*

#### Guidance in ACP

Several interviewees were unfamiliar with the term “advance care planning” prior to the interview and these interviewees would rather search for information about health related problems or their disease. However, most interviewees mentioned to have experience with ACP related topics such as discussing preferences with relatives and healthcare professionals, and thinking about preferred treatment and care in the future. Those who were familiar, would search for information about the concept of ACP and why it is important. The interviewees stressed the importance of adapting ACP to individual needs, since patients’ coping styles differ, and patients have different cultural backgrounds. Many interviewees considered it important to be encouraged by the information, their healthcare providers or relatives to think about ACP, to take a pro-active role in ACP, and to take responsibility for their health and healthcare. Some interviewees preferred information about rules and instructions for the completion of advance directives and several interviewees mentioned the importance of the revision of preferences over time. Search terms related to this information need included: “Advance care planning”; “What is ACP”; the Dutch term “Vroegtijdige zorgplanning”; “Care in the future”; “How can I control my care?”; “Prepare for the future”; “Recording of wishes”; and “Advance Directive”.

*Patient 4 (age 42), subtheme “Importance of being encouraged to do ACP and to take a pro-active role in ACP”: “I took the initiative to start the conversation with my general practitioner about my treatment and care preferences. Yes, I think that you have to start this [ACP] from your own motivation. Because you have to come up with these preferences on your own. That it is something you have to think about independently. Yes, what if… yes, what do I want? What do I not want? And that is a process, you do not know that the first time. You don't know that in the first week, you may not even know that in the first year.”*

#### How to communicate treatment and care preferences

The interviewees preferred information on how to involve family in ACP, and how to start conversations with family and with healthcare professionals. Some interviewees mentioned that patients should be encouraged to start these conversations. Some interviewees wanted information about rules and instructions for the appointment of a healthcare representative.

*Patient 9 (age 53), subtheme “Tips to start the conversation with healthcare professionals”: “Such a website should come up with a number of example questions. Namely, if you want to have a care planning meeting with your doctor, there is a certain process, for example if it is about whether you have to undergo surgery. A number of example questions on such a website would help people. I know there are health insurance companies that make lists of questions you can write down before visiting your doctor, which is very useful. It often happens to me that I forget to ask important questions to my doctor.”*

#### Peer support and sharing experiences

The interviewees considered peer support and experience stories as helpful. The interviewees would also search for relevant patient associations to find practical- and peer support in their disease and care.

*Patient 9 (age 53), subthemes “Information about patient associations” and “Information about peer support”: “Well, in my case, I just started to search for information at websites of [two disease specific] patient associations and started to look from there. Those were two main sources for me. At the [third disease specific] patient association, I started to look further and then I also looked for peer support.”*

#### Information for relatives

The interviewees (patients as well as relatives) also preferred information for relatives, for example on how they could support their chronically ill relative. Search terms were “How does the family cope with illness of a relative?” and “What is the impact of [the disease] on the family?”

*Relative 3 (age 62), main theme “Information for relatives”: “Well, in addition to all the information that is available for patients... it is also important for me as a relative, to know where I can go if I experience problems and my partner or child isn't open to discuss these. I would like to receive information about how I can cope with the situation and what I can do, and receive contact information about where I can go if I can’t take it anymore.”*

**Fig. 1:**
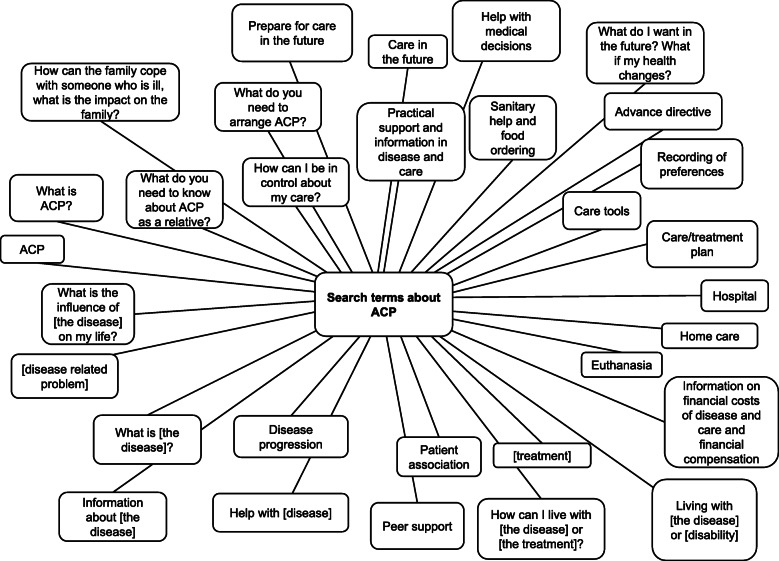
Search terms on the web that patients and relatives use to find ACP related information

All mentioned search terms that the interviewees would use while searching ACP information on the Internet, are presented in Fig. 1.

## Discussion

The interviewees preferred information about their disease trajectory and quality of life, about medical treatment decisions, and about practical support in arranging their care. Furthermore, they appreciated information about ACP and guidance in ACP, about communication of treatment and care preferences and about peer support of others with chronic diseases. Lastly, they appreciated information for relatives.

Many of the mentioned ACP related topics correspond with the key elements for ACP as formulated by an international taskforce of ACP experts [1]. Overlapping topics relate to the recording of ACP and the appointment of a healthcare representative. However, many new information needs emerged, such as the need to take a pro-active role in ACP, the need for peer support and information about patient associations, the need for practical support with disease and care and the need for information for relatives. Also, not all interviewees were aware of the concept of ACP. This indicates that patients and their relatives may need more information than currently recommended by healthcare professionals [1].

One topic that emerged, which was yet undescribed in the ACP key elements [1], is the encouragement to start with ACP and taking a pro-active role, which the interviewees considered important. Reports about patients’ willingness to initiate ACP differ. For instance, Bernacki et al. [17] describe that patients expect clinicians to initiate ACP, and Jabbarian et al. [3] describe that patients and healthcare professionals perceive each other as being reluctant to initiate ACP. Similar to the findings of Zwakman et al. [18] that patients differ in readiness and willingness to be open in ACP, the current study found that individual needs in ACP, and the preference to initiate ACP may differ from person to person. Therefore, we recommend to take into account that some, but not necessarily all patients may prefer a pro-active role in ACP. This could be done by adapting ACP to a patient’s readiness to engage in ACP, to their disease stage, and to legal and cultural circumstances [1].

This study contributes to the small number of studies related to the topic of patients’ and relatives’ information needs for ACP [4, 7] as well as to the rarely studied topic of information needs for web-based ACP. This study provided insight in ACP information needs from the perspective of patients and relatives.

A varied sample of interviewees was selected with different diagnoses, relationships to relatives, and ages. The sample in this explorative study was relatively small, but saturation was reached.

This study showed that patients with chronic disease and their relatives need reliable information about ACP, their health condition, treatment and care, and about communication and peer support. We took these information needs into account during the development of the Dutch web-based ACP program: “Verken uw wensen voor zorg en behandeling” (English translation: “Explore your preferences for treatment and care” [19]. The program is embedded in the website “www.thuisarts.nl” (English version: “www.GPinfo.nl”), which is developed by general practitioners and contains reliable information about health, diseases, care and treatments. The web-based program is interactive as it guides users through the ACP process step by step; it helps them to consider, discuss and record treatment and care preferences. Users can answer questions, watch videos, and print a document with their answers to the questions asked in the program. The web-based program refers to information on other webpages of the “www.thuisarts.nl” website, and it also contains hyperlinks to patient organizations, in concordance with the needs of patients and their relatives. Based on the findings of this study which shows that patients and their relatives need information on a broad range of topics and not only on ACP specifically, we think the web-based ACP program is a valuable tool to patients and their relatives.

The information needs of patients and their relatives for ACP presented in this study, can also be used as input in the development of other new ACP interventions.

## Conclusions

In conclusion, information needs for ACP included guidance in ACP, support in making decisions about medical treatment, and practical support in arranging care. Not all interviewees were aware of the concept of ACP. We recommend adapting web-based ACP information to the information needs and search terms of patients and their relatives to increase its findability, uptake and usefulness for those who want to engage in ACP.

## Supplementary Information

**Additional file 1:** **Appendix A.** Coding tree.

## Data Availability

The datasets generated and/or analysed during the current study are not publicly available because the data are confidential, but are available from the corresponding author on reasonable request.

## Abbreviations

ACP: Advance care planning
ALS: Amyotrophic lateral sclerosis
COPD: Chronic obstructive pulmonary disease
MS: Multiple sclerosis
COREQ: COmprehensive consolidated criteria for REporting Qualitative research
