# Supplementary figures and images for: Information needs of patients with chronic diseases and their relatives for web-based advance care planning: a qualitative interview study
Source: BMC Palliat Care. 2021 May 30;20:77. doi: 10.1186/s12904-021-00770-x (PMC8164830; doi:10.1186/s12904-021-00770-x)

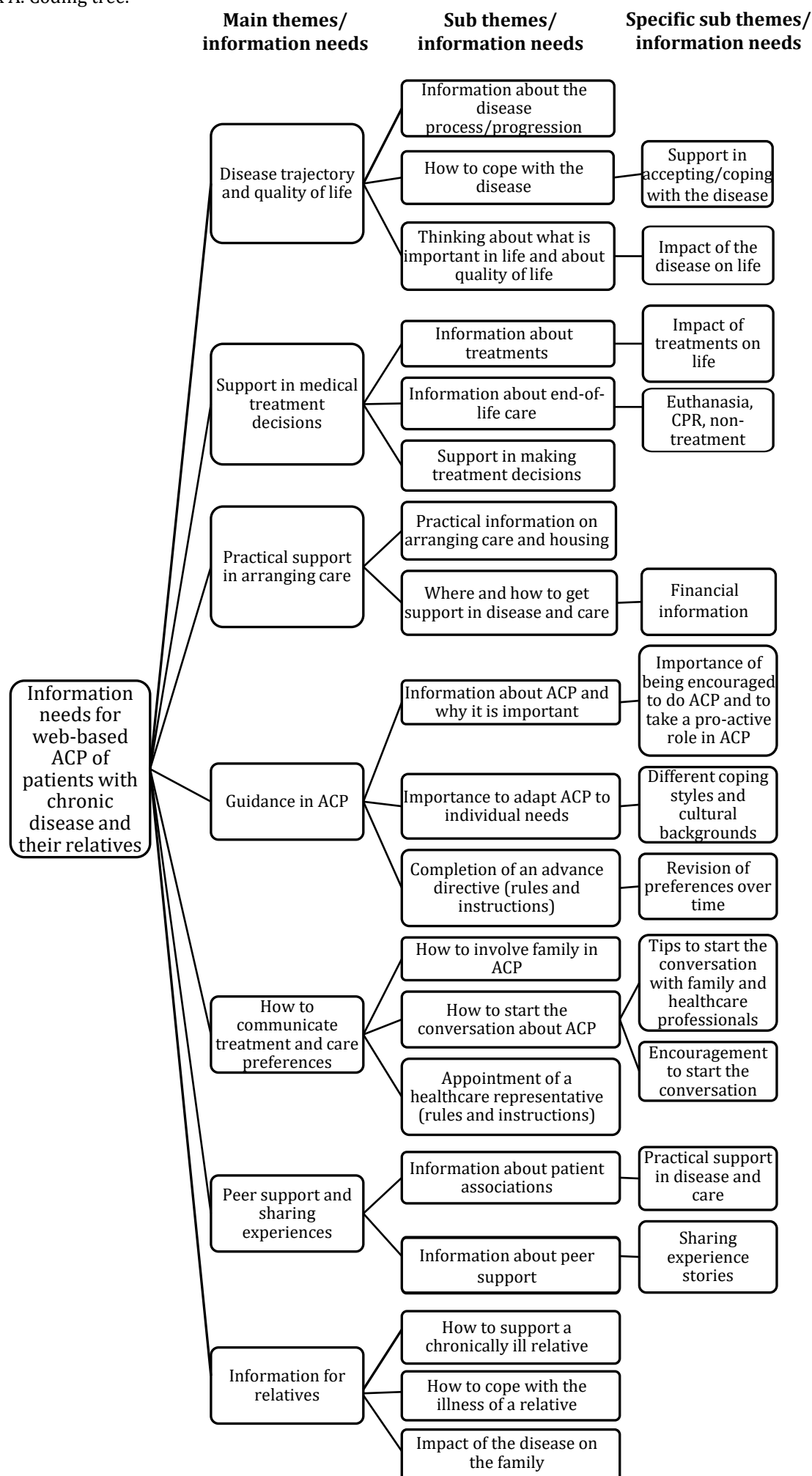

Supplement: Supplementary file 1 — Additional file 1: Appendix A. Coding tree. [file 12904_2021_770_MOESM1_ESM.pdf]
